# Cracking the superheavy pyrite enigma: possible roles of volatile organosulfur compound emission

**DOI:** 10.1093/nsr/nwab034

**Published:** 2021-03-01

**Authors:** Xianguo Lang, Zhouqiao Zhao, Haoran Ma, Kangjun Huang, Songzhuo Li, Chuanming Zhou, Shuhai Xiao, Yongbo Peng, Yonggang Liu, Wenbo Tang, Bing Shen

**Affiliations:** State Key Laboratory of Oil and Gas Reservoir Geology and Exploitation, and Institute of Sedimentary Geology, Chengdu University of Technology, Chengdu 610059, China; Key Laboratory of Orogenic Belts and Crustal Evolution of the Ministry of Education, and School of Earth and Space Science, Peking University, Beijing 100871, China; State Key Laboratory of Palaeobiology and Stratigraphy, Nanjing Institute of Geology and Palaeontology, and Center for Excellence in Life and Palaeoenvironment, Chinese Academy of Sciences, Nanjing 210008, China; Key Laboratory of Orogenic Belts and Crustal Evolution of the Ministry of Education, and School of Earth and Space Science, Peking University, Beijing 100871, China; School of Physics, Peking University, Beijing 100871, China; Key Laboratory of Orogenic Belts and Crustal Evolution of the Ministry of Education, and School of Earth and Space Science, Peking University, Beijing 100871, China; State Key Laboratory of Continental Dynamics, Northwest University, Xi'an 710069, China; Shaanxi Key Laboratory of Early Life and Environments, Department of Geology, Northwest University, Xi’an 710069, China; State Key Laboratory of Oil and Gas Reservoir Geology and Exploitation, and Institute of Sedimentary Geology, Chengdu University of Technology, Chengdu 610059, China; State Key Laboratory of Palaeobiology and Stratigraphy, Nanjing Institute of Geology and Palaeontology, and Center for Excellence in Life and Palaeoenvironment, Chinese Academy of Sciences, Nanjing 210008, China; Department of Geosciences, Virginia Polytechnic Institute and State University, Blacksburg, VA 24061, USA; International Center for Isotope Effect Research, Nanjing University, Nanjing 210023, China; School of Earth Sciences and Engineering, Nanjing University, Nanjing 210023, China; School of Physics, Peking University, Beijing 100871, China; School of Mathematical and Statistical Sciences, Arizona State University, Tempe, AZ 85287, USA; Key Laboratory of Orogenic Belts and Crustal Evolution of the Ministry of Education, and School of Earth and Space Science, Peking University, Beijing 100871, China

**Keywords:** sulfur isotope, organosulfur compound, sulfur cycle, Cryogenian, Datangpo Formation

## Abstract

The global deposition of superheavy pyrite (pyrite isotopically heavier than coeval seawater sulfate in the Neoproterozoic Era and particularly in the Cryogenian Period) defies explanation using the canonical marine sulfur cycle system. Here we report petrographic and sulfur isotopic data (δ^34^S_py_) of superheavy pyrite from the Cryogenian Datangpo Formation (660–650 Ma) in South China. Our data indicate a syndepositional/early diagenetic origin of the Datangpo superheavy pyrite, with ^34^S-enriched H_2_S supplied from sulfidic (H_2_S rich) seawater. Instructed by a novel sulfur-cycling model, we propose that the emission of ^34^S-depleted volatile organosulfur compounds (VOSC) that were generated via sulfide methylation may have contributed to the formation of ^34^S-enriched sulfidic seawater and superheavy pyrite. The global emission of VOSC may be attributed to enhanced organic matter production after the Sturtian glaciation in the context of widespread sulfidic conditions. These findings demonstrate that VOSC cycling is an important component of the sulfur cycle in Proterozoic oceans.

## Teaser

This paper presents a new marine sulfur-cycling model that quantitatively describes the worldwide deposition of superheavy pyrite after the Neoproterozoic Snowball Earth glaciation.

## INTRODUCTION

The marine sulfur biogeochemical cycle is closely tied to the global carbon cycle via dissimilatory microbial sulfate reduction (MSR), in which sulfate (SO_4_^2−^) is reduced to hydrogen sulfide (H_2_S) using organic matter (CH_2_O) as an electron donor [1,2]. Because sulfate-reducing microbes preferentially utilize ^32^S-enriched sulfate, H_2_S is always depleted in ^34^S as compared with sulfate [3–6]. Sequestration of H_2_S by reaction with reactive Fe in seawater/sediments results in the precipitation of syngenetic (i.e. direct precipitation from seawater) or diagenetic (i.e. formed in sediment porewater) pyrite (FeS_2_), which is the predominant sulfide mineral in sediments and sedimentary rocks. Because of negligible isotopic fractionation (<3‰) in pyrite formation [7], pyrite sulfur isotopic values (δ^34^S_py_) are expected to be lower than that of contemporaneous seawater sulfate (δ^34^S_sw_).

**Figure 1.**
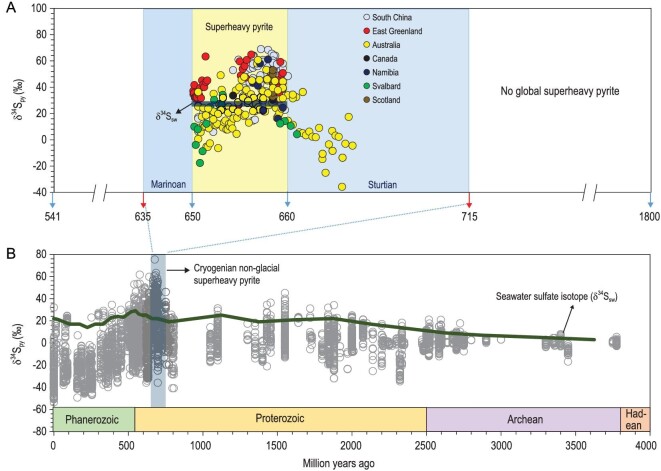
A compilation of δ^34^S_py_ data showing superheavy pyrite in Earth history. (A) is an expanded view of (B), modified from Canfield and Farquhar [16], highlighting superheavy pyrite from the Cryogenian non-glacial interval in South China [11,13,21,22], East Greenland [14], Australia [9], Canada [9], Namibia [17,37], Svalbard [39] and Scotland [38]. Seawater sulfate isotope values (δ^34^S_sw_), green line in (A), are estimated from sulfur isotopic compositions of evaporates in Australia [9].

However, superheavy pyrite, referring to pyrite with δ^34^S_py_ > δ^34^S_sw_, has been widely reported from ancient sedimentary rocks and modern marine sediments [8]. Although pyrite with high δ^34^S_py_ value can be explained by MSR in a close system through Rayleigh distillation process, superheavy pyrite with high abundances (>0.1 wt.%), prolonged stratigraphic occurrences (>1 million years) and global distributions [9–14] cannot be attributed to MSR and pyrite formation in a close system [15]. For example, superheavy pyrite is particularly abundant in deposits of the Cryogenian non-glacial interval (660–650 Ma) (Fig. 1A), where δ^34^S_py_ values can be as high as +80‰, much higher than the non-glacial δ^34^S_sw_ value of ∼+30‰ [9,16,17]. Another geological interval characterized by global occurrence of superheavy pyrite is the late Cambrian Steptoean Positive Carbon Isotope Excursion (SPICE) event (∼499 Ma) with δ^34^S_py_ values up to +70‰ [18], although the duration of SPICE is significantly shorter (∼1 Myr). Several models have been proposed to explain the precipitation of superheavy pyrite, including sulfide oxidation, fast sedimentation rate, thermogenesis, oceanic anoxia and carbon-sulfur cycle models [18–23]. However, these models do not satisfactorily address the high abundance and global occurrence of superheavy pyrite in the Cryogenian non-glacial interval.

Most previous investigations of superheavy pyrite were based on geochemical data, but did not fully consider petrographic data, which are critical in understanding the environmental context of pyrite formation. For example, the size distributions of framboidal and euhedral pyrite can be used to infer marine redox conditions [24]. With few exceptions [21], such petrographic data (e.g. framboidal pyrite vs. euhedral pyrite; size distribution of pyrite) were not integrated in the interpretation of superheavy pyrite. The lack of sedimentological and petrographic contexts makes it difficult to assess the various models of superheavy pyrite formation.

It is widely accepted that organosulfur compounds play an important role in the modern marine sulfur cycle [25]. Some of the simplest yet most common organosulfur compounds are methanethiol (CH_3_SH or MeSH) and dimethyl sulfide (CH_3_SCH_3_ or DMS) [26], both of which are highly volatile and accordingly termed the volatile organosulfur compounds (VOSC). VOSC can be produced in two different ways. First, VOSC can be generated through the dimethylsulfoniopropionate (DMSP) pathway in surface ocean (DMSP-derived VOSC), in which VOSC derives from the degradation of DMSP, a product of phytoplankton [27]. It is estimated that the annual DMSP-derived VOSC flux is ∼1 Tmol, which is one-third of the riverine sulfate input (∼3 Tmol yr^−1^) [27]. Because there is little sulfur isotope fractionation during VOSC formation and emission [28], DMSP-derived VOSC does not change δ^34^S_sw_. Alternatively, VOSC can be generated through H_2_S methylation (H_2_S-derived VOSC) in the presence of H_2_S, e.g. in sulfidic water. H_2_S-derived VOSC inherits the isotope signal of H_2_S [28] and thus always has lower δ^34^S values relative to δ^34^S_sw_ [28]. H_2_S methylation is expected to have been an important part of the sulfur cycle in sulfidic Proterozoic oceans [29]. Sustained emission of ^34^S-depleted VOSC would thus elevate the δ^34^S of the residual sulfur pool of sulfidic seawater. We suggest that the interpretation of superheavy pyrite in the geological record should consider the organosulfur cycle.

In this study, we first reviewed the occurrences of superheavy pyrite during the Cryogenian non-glacial interval. Then we presented (i) detailed petrographic observations and (ii) paired pyrite content and sulfur isotope data of superheavy pyrite from the Cryogenian non-glacial deposits of the Datangpo Formation in South China (Figs S1 and S2). Based on the petrographic and geochemical data, the existing models were evaluated, and a quantitative analysis was applied to simulate the process of superheavy pyrite formation. Finally, a novel sulfur cycle model that incorporates organosulfur compounds was proposed to interpret the superheavy pyrite formation.

## GEOLOGICAL RECORD OF SUPERHEAVY PYRITE

Compilation of pyrite sulfur isotope data shows that superheavy pyrite occurs throughout the Earth's history (Fig. 1), including Mesoproterozoic, Cryogenian non-glacial interval, Ediacaran, Cambrian, Ordovician, Devonian, Triassic, Cretaceous, Pleistocene and even modern marine sediments[16–18,20,23,30–33]. Below, we briefly review the geological records of superheavy pyrite.

It is not uncommon to find individual pyrite crystal with extremely high δ^34^S_py_ value in either sedimentary rocks or sediments of any age. For example, individual micrometer-sized pyrite crystals from late Cretaceous sediments show a wide range of δ^34^S_py_ values, with the highest value of +89.3‰ [34], and δ^34^S_py_ of pyrite crystals from modern marine sediments can be more than +30‰ [8]. On the other hand, many Proterozoic sedimentary rocks, such as the Mesoproterozoic Chuanlinggou Formation in North China [30], Ediacaran Doushantuo Formation in Yangtze Block [35] and Ediacaran Nama Group in Namibia [36], have bulk-sample δ^34^S_py_ values greater than +30‰ (the expected upper bound of Proterozoic δ^34^S_sw_). It should be noted that, in these studies, the bulk-sample pyrite contents are typically very low (<0.1 wt.%), and there is no consistent stratigraphic occurrence.

Although pyrite is not uncommon within the geological record, the sustained precipitation of superheavy pyrite at a global scale and for millions of years was rare. There are two geological intervals characterized by worldwide distributions of superheavy pyrite, and these superheavy pyrite windows include the Cryogenian non-glacial interval (Fig. 1A) and the Cambrian SPICE event. Unlike other geological intervals with sporadically occurring superheavy pyrite, these two intervals are characterized by superheavy pyrite with consistent stratigraphic occurrences (>1 Myr), global occurrence and high pyrite content (>1 wt.%) [13,18]. In the case of the Cryogenian non-glacial interval, δ^34^S_py_ shows a clear decreasing trend from ca. +80‰ to ca. +30‰ within 10 million years (Fig. 1A). In the SPICE event, δ^34^S_py_ shows a prominent positive excursion to the maximum value of ∼+70‰, and this positive excursion can be correlated globally as well [18].

**Figure 2.**
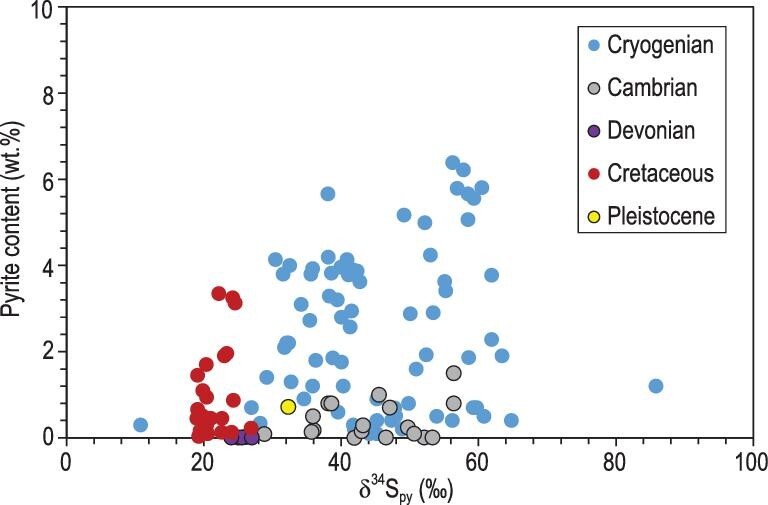
δ^34^S_py_ and pyrite content of ancient superheavy pyrite in fine-grained sediments of the Cryogenian [11,13], Cambrian [18], Devonian [32], Cretaceous [31,33] and Pleistocene [19]. In contrast with superheavy pyrite of other ages, Cryogenian samples have higher contents of superheavy pyrite.

The Cryogenian non-glacial interlude might be the only period in Earth's history with global occurrence of superheavy pyrite for over ∼10 million years. Superheavy pyrite of this geological interval has been reported from South China [11–13], Namibia [37], East Greenland [14], Scotland [38], Svalbard [39], Australia and Canada [37,40] (Fig. 1A). Bulk-sample δ^34^S_py_ values are normally greater than +30‰ with extreme values up to +80‰ (Fig. 1A). Although the δ^34^S_sw_ of this interval has not been systematically investigated, these δ^34^S_py_ values are higher than the sulfur isotopic values of gypsum from coeval deposits in Australia that range from +26‰ to +30‰ [40]. Because isotope fractionation during evaporate precipitation is negligible [41], these gypsum values can be taken as δ^34^S_sw_ values. If so, δ^34^S_py_ of Cryogenian superheavy pyrite obviously exceeds contemporaneous δ^34^S_sw_ values and cannot be explained by microbial sulfate reduction alone, particularly considering that these samples typically have high pyrite content (>0.1 wt.%) [13] (Fig. 2) and that δ^34^S_py_ values appear to show a spatial gradient, with higher δ^34^S_py_ values in deep water sections [13,22].

## PETROGRAPHY AND GEOCHEMISTRY OF SUPERHEAVY PYRITE IN THE DATANGPO FORMATION

To provide further insights into the origin of Cryogenian superheavy pyrite, we investigate the petrography and geochemistry of superheavy pyrite from the Datangpo Formation in the Yangtze Block, South China. The Datangpo Formation is precisely dated between ∼660 Ma and ∼650 Ma [42,43], and represents the deposition in the Cryogenian non-glacial interval in South China. Samples were collected from two drill cores (ZK-WL and ZK-DL) in northeast Guizhou Province. In both drill cores, the Datangpo Formation begins with an Mn-rich carbonate of several meters in thickness, followed by black shale of several tens of meters in thickness. Paleogeographic reconstruction indicates that both sections were located in the continental slope of the Yangtze Block in late Neoproterozoic [44] (Figs S1 and S2).

Three types of pyrite are observed in the Datangpo Formation: pyrite nodules, pyrite laminae and disseminated pyrite (Fig. S3). Both pyrite nodules and laminae occur sporadically, but disseminated pyrite is present abundantly throughout the Datangpo Formation. The disseminated pyrite includes both euhedral and framboidal pyrite. Framboidal pyrite is composed of densely packed, spheroidal pyrite crystallites of sub-micron size (Fig. S3), and ranges from 1 μm to 12 μm in diameter (Fig. S4). In contrast, rhombic euhedral pyrite crystals are less common, but they range from 100 μm to 200 μm in size (Fig. S3). Therefore, although pyrite framboids are more numerous, euhedral pyrite accounts for ∼90% (mean = 89.7%, *n* = 23) of total pyrite in mass (Table S1).

**Figure 3.**
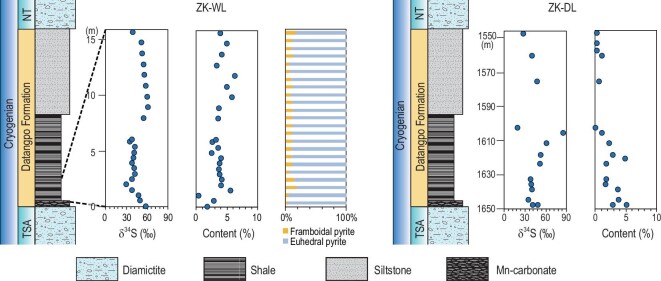
δ^34^S_py_, pyrite content and pyrite morphologies of Cryogenian non-glacial deposits in the Datangpo Formation at ZK-WL and ZK-DL drill cores in South China.

Most δ^34^S_py_ values of the Datangpo Formation are greater than +40‰, with an average value of +45.4‰ (*n* = 43) (Fig. 3 and Table S2). The bulk-sample pyrite content varies between 0.1 wt.% and 6.4 wt.%, with a mean value of 3.3 wt.% (*n* = 43) (Fig. 3 and Table S2). In both drill cores, δ^34^S_py_ values and pyrite contents show a decreasing trend. At ZK-DL, the lower 40 m interval of the Datangpo Formation has an average δ^34^S_py_ value of +46.7‰ and an average pyrite content of 3.9 wt.%, whereas the upper part of the Formation has lower δ^34^S_py_ values (mean = +37.2‰) and pyrite contents (mean = 0.6 wt.%). At ZK-WL, the Datangpo Formation has an average δ^34^S_py_ value of +46.8‰ and pyrite content of 3.8 wt.% (Table S2).

## SULFUR SOURCE OF DATANGPO SUPERHEAVY PYRITE

Predominant euhedral pyrite (>80%, Table S1) in the Datangpo Formation suggests a diagenetic origin in sediment porewater or near sediment-water interface (SWI). Pyrite formation could be fueled by MSR in porewater, or H_2_S diffusion from sulfidic seawater [15]. In the former case, δ^34^S_py_ can be simulated by the Rayleigh distillation model, if a closed system can be assumed, or by the sulfate diffusion-advection-reaction (DAR) model in an open system [15].

Rayleigh distillation in a closed system can only produce high instantaneous δ^34^S values during the terminal stage, at which time porewater sulfate is almost quantitatively converted to pyrite and the cumulative δ^34^S_py_ value approaches δ^34^S_sw_. However, cumulative δ^34^S_py_ values of superheavy pyrite, as represented by bulk-sample measurements from the Datangpo Formation, are up to +70‰, notably higher than coeval δ^34^S_sw_ (+30‰). Therefore, Rayleigh distillation cannot fully account for the Datangpo data reported here.

Alternatively, in an open system, porewater sulfate isotopic composition can be calculated by the DAR model. The sulfate and sulfide profiles can be calculated by

(1) \begin{eqnarray*} \frac{{\partial \left[ {{}_{\ }^iSO_4^{2 - }} \right]}}{{\partial t}} &=& {D_{\rm s}} \!\left( {\frac{{{\partial ^2}\left[ {{}_{\ }^iSO_4^{2 - }} \right]}}{{\partial {z^2}}}} \right)\nonumber\\ -\,\, s \! \left({\frac{{\partial \left[ {{}_{\ }^iSO_4^{2 - }} \right]}}{{\partial z}}} \right)\nonumber\\ -\,\, R_{\rm MSR}^i\left[ {{}_{\ }^iSO_4^{2 - }} \right]\left[ {C{H_2}O} \right],\nonumber\\ \end{eqnarray*}

and

(2) \begin{eqnarray*} \frac{{\partial \left[ {{H_2}{}_{\rm{\ }}^iS} \right]}}{{\partial t}} &=& {D_{\rm {H_2}S}} \! \left({\frac{{{\partial ^2} \!\left[{{H_2}{}_{\rm{\ }}^iS} \right]}}{{\partial {z^2}}}} \right)\nonumber\\ -\,\, s \!\left( {\frac{{\partial \!\left[{{H_2}{}_{\rm{\ }}^iS} \right]}}{{\partial z}}} \right)\nonumber\\ +\,\, R_{\rm MSR}^i\left[ {{}_{\rm{\ }}^iSO_4^{2 - }} \right]\left[ {C{H_2}O} \right]\nonumber\\ -\,\, R_{\rm py}^i\left[ {{H_2}{}_{\rm{\ }}^iS} \right]\left[ {Fe} \right],\end{eqnarray*}

respectively. Here, ${D_s}$ and ${D_{\rm {H_2}S}}$ are porewater sulfate and H_2_S diffusion coefficients, *s* is sedimentation rate, $[ {C{H_2}O} ]$ is organic matter content, $R_{\rm MSR}^i$ and $R_{\rm py}^i$ are reaction rate constants for MSR and pyrite formation respectively, and ‘*i*’ is 32 or 34.

MSR and pyrite formation consume organic matter and reactive iron. Their concentration profiles in sediments can be expressed by

(3) \begin{eqnarray*} \frac{{\partial \!\left[{C{H_2}O} \right]}}{{\partial t}} &=& - s \!\left({\frac{{\partial \!\left[ {C{H_2}O} \right]}}{{\partial z}}} \right) \nonumber\\ -\,\, R_{\rm MSR}^{32}\left[ {{}_{\ }^{32}SO_4^{2 - }} \right]\left[ {C{H_2}O} \right]\nonumber\\ -\,\, R_{\rm MSR}^{34}\left[ {{}_{\ }^{34}SO_4^{2 - }} \right]\left[ {C{H_2}O} \right],\nonumber\\ \end{eqnarray*}

and

(4) \begin{eqnarray*}\frac{{\partial \left[ {Fe} \right]}}{{\partial t}} &=& - s \!\left({\frac{{\partial \left[ {Fe} \right]}}{{\partial z}}} \right) - R_{\rm py}^{32}\left[ {{H_2}{}_{\rm{\ }}^{32}S} \right]\left[ {Fe} \right]\nonumber\\ -\,\, R_{\rm py}^{34}\left[ {{H_2}{}_{\rm{\ }}^{34}S} \right]\left[ {Fe} \right].\end{eqnarray*}

Combining equations (1) to (4), ${\rm{\delta }}{}_{\ }^{34}{{\rm{S}}_{\rm py}}$ and pyrite content can be calculated as follows:

(5) \begin{eqnarray*} {\rm{\delta }}{}_{\ }^{34}{{\rm{S}}_{\rm py}} &=& {\rm{ln}}\bigg( \frac{{\smallint _0^zR_{\rm py}^{34}\left[ {{H_2}{}_{\ }^{34}S} \right]\left[ {Fe} \right]dz}}{{\smallint _0^zR_{\rm py}^{32}\left[ {{H_2}{}_{\ }^{32}S} \right]\left[ {Fe} \right]dz}}\bigg/\nonumber\\ \quad \frac{{{}_{\ }^{34}{S_{\textit{standard}}}}}{{{}_{\ }^{32}{S_{\textit{standard}}}}} \bigg) \times 1000\,{^{\circ}/{\circ\circ}}, \end{eqnarray*}

(6) \begin{eqnarray*} \left[{\textit{pyrite}} \right]\! \left( \% \right) &=& \frac{{{M_{\rm p y}} \times \varphi }}{{2 \times s \times \left( {1 - \varphi } \right) \times \rho }}\nonumber\\ \cdot\mathop \int \nolimits_0^z (R_{\rm p y}^{32}\left[ {{H_2}{}_{\ }^{32}S} \right]\left[ {F\! e} \right]\nonumber\\ +\,\, R_{\rm py}^{34}\left[ {{H_2}{}_{\ }^{34}S} \right]\left[ {F\! e} \right])dz.\nonumber\\ \end{eqnarray*}

**Figure 4.**
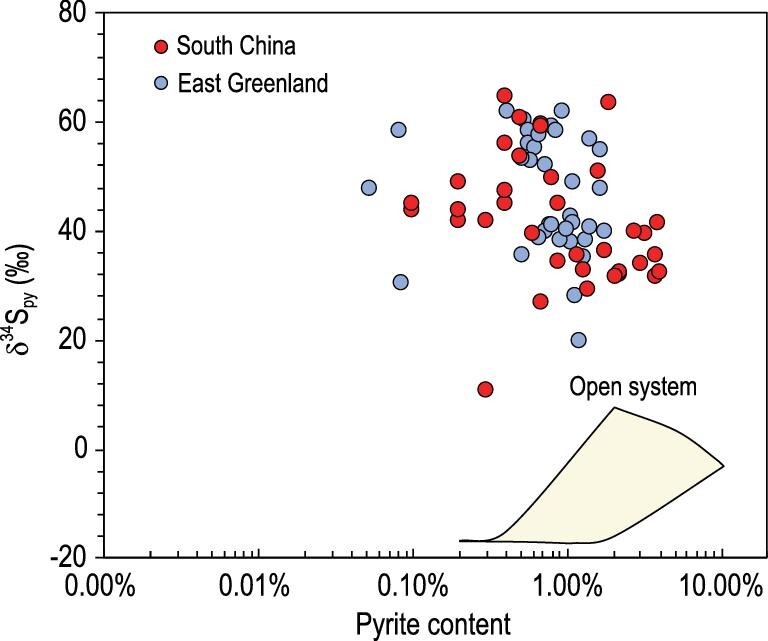
δ^34^S_py_ and pyrite content data from the Cryogenian non-glacial interval in South China and East Greenland. Also shown are 1D-DAR model results (yellow area) depicting the relationship between δ^34^S_py_ and pyrite content when MSR occurs in an open system. In this model, sulfate diffusion coefficient (*D*s), MSR-associated sulfur isotope fractionation, seawater sulfate concentration and sedimentation rate are set at 3.61 × 10^−6^ cm^2^ s^−1^, 47‰, 5 mM L^−1^, and 0.04 cm yr^−1^, respectively, whereas $R_{\rm py}^{32}$ varies from 10^−3^ yr^−1^ to 25 yr^−1^.

Organic matter content ([*CH_2_O*]) is set between 1.25 wt.% and 12.5 wt.%. Sulfate diffusion coefficient (*D*_s_), sulfate concentration and sedimentation rate (*s*) are 3.61 × 10^−6^ cm^2^ s^−1^ [45], 5 mM L^−1^ [46], and 0.05 cm ky^−1^ (based on the maximum thickness of 500 m and a duration time of 10 Myr), respectively. The modeling results indicate that high δ^34^S_py_ values cannot be simulated by the sulfate-DAR model by MSR in sediment porewater with sulfate diffusion from seawater (Fig. 4).

Therefore, neither the Rayleigh distillation model nor the sulfate-DAR model can explain the coupled high δ^34^S_py_ values and high pyrite contents of the Datangpo Formation (Fig. 4), suggesting that the Datangpo pyrite cannot be generated by MSR in sediment porewater. Below, we briefly explore the possibility that the H_2_S for superheavy pyrite formation in the Datangpo Formation may have been sourced from sulfidic seawater.

Because of the small isotopic fractionation associated with pyrite precipitation from H_2_S and Fe^2+^, δ^34^S_py_ values are mainly determined by the isotopic composition of H_2_S in sulfidic seawater (δ^34^S_H2S_). High δ^34^S_H2S_ requires high fraction of seawater sulfate reduction. On the other hand, bulk-sample pyrite content is controlled by many factors, including seawater H_2_S concentration, reaction rate of pyrite formation and sedimentation rate. High seawater H_2_S concentration and slow sedimentation rate would lead to high pyrite content. Thus, high δ^34^S_py_ values and a high pyrite content of the Datangpo superheavy pyrite may result from a high fraction of sulfate reduction in sulfidic seawater, slow precipitation rate and fast pyrite precipitation in sediment porewater.

The development of oceanic euxinia is consistent with the widespread distribution of framboidal pyrite in the Datangpo Formation (Fig. S3), although it is quantitatively less important than euhedral pyrite. It is also consistent with Fe speciation data that show high Fe_py_/Fe_HR_ ratios (>0.8) [13], as well as low Mo concentrations in black shale [13,14].

Pyrite from the Cryogenian non-glacial interval is also characterized by both spatial and temporal patterns. For example, pyrite from the slope sections is more enriched in ^34^S than that from the shelf sections, possibly indicating a δ^34^S_H2S_ gradient in the Yangtze Block [13,22]. Moreover, the compilation of global data indicates a decreasing trend of δ^34^S_py_ values from the earlier to the later part of the Cryogenian non-glacial interval (Fig. 1), suggesting the secular pattern of seawater δ^34^S_H2S_. Therefore, the degree of sulfate reduction in sulfidic seawaters may have had systematic temporal and spatial patterns in the Cryogenian non-glacial interval. Below, we first evaluate previously published hypotheses about the origin of superheavy pyrite, followed by a quantitative model that incorporates VOSC to account for the observed spatial pattern of δ^34^S_py_.

## EVALUATING SUPERHEAVY PYRITE MODELS

Several models have been proposed to interpret superheavy pyrite formation, including the sulfide oxidation [20,23], high sedimentation rate [19], thermogenesis [21,22], ocean anoxia [13,23,47,48] and carbon-sulfur cycle models [13,18,47,48]. Based on the petrographic and geochemical analyses of the Datangpo superheavy pyrite, we argue that a successful model needs to explain the following observations and inferences about Cryogenian superheavy pyrite: (i) the global distribution and persistent stratigraphic occurrence, (ii) high pyrite contents, (iii) precipitation in sediment or near SWI, and (iv) the spatial gradient and temporal variation of δ^34^S_py_. Below we evaluate whether the existing models can explain these observations.

### Sulfide oxidation model

The sulfide oxidation model was originally proposed to interpret superheavy pyrite in the late Ediacaran Nama Group [20]. In this model, the preferential aerobic oxidation of ^32^S-enriched H_2_S results in ^34^S-enrichment of the remaining H_2_S and precipitation of pyrite with high δ^34^S_py_ values [20,23]. Experimental studies indicate that isotopic fractionations of 4‰ and 18‰ are associated with inorganic and biological H_2_S oxidation, respectively [49]. Based on this model, high δ^34^S_py_ values also require low seawater sulfate concentration that limits isotope fractionation in MSR [20]. The sulfide oxidation model can be applied to the Nama superheavy pyrite, because widespread storm deposits in the Nama Group indicate active sulfide oxidation in a well-mixed ocean [20]. In addition, generation of high δ^34^S_py_ values through sulfide oxidation also implies high fraction of sulfide oxidation, resulting in low pyrite contents. Thus, the sulfide oxidation model can explain superheavy pyrite with low pyrite content from high-energy depositional settings, e.g. shallow marine settings above the fair-weather wave base. However, the Datangpo Formation is composed of black shale and fine-laminated siltstone with abundant framboidal pyrite (Figs S2 and S3). The absence of ripple marks or cross-bedding structures indicates deposition below the fair-weather wave base. This sedimentological interpretation is also consistent with Fe speciation data, redox sensitive trace element compositions (e.g. Mo) and sulfur isotopes of the Datangpo samples [13,50], arguing against the sulfide oxidation model.

### Fast sedimentation rate model

The fast sedimentation model was initially proposed to explain superheavy pyrite in deposits of the Last Glacial period with a high sedimentation rate ranging from 50 cm kyr^−1^ to 250 cm kyr^−1^ [19]. Sedimentary pyrite from coarse-grained sediments has higher δ^34^S_py_ values relative to those from fine-grained sediments, because high sedimentation rates limit the exchange between sediment porewater and seawater [19], thus limiting the supply of seawater sulfate for MSR. Quantitative reduction of porewater sulfate at fast sediment rates would limit isotopic fractionation in MSR and hence lead to high δ^34^S_py_ values (approaching a closed system) [45]. Although it remains unclear how δ^34^S_py_ can exceed δ^34^S_sw_, some superheavy pyrite crystals are indeed discovered in glacial deposits with high sedimentation rates [19]. This model may explain superheavy pyrite occurring sporadically in settings with a fast sedimentation rate, such as shoreface or delta deposition environments with coarse-grained sediments.

We argue, however, that fast sedimentation rate alone cannot explain the superheavy pyrite in the Datangpo Formation, because it is mainly composed of black shale and laminated muddy siltstone (Figs S2 and S3), which likely record slow sedimentation rates. A rough estimate of sedimentation rate of the Datangpo Formation ranges from 0.1 cm kyr^−1^ to 5 cm kyr^−1^ (based on the stratigraphic thickness of 10–500 m and the duration of 10 Myr of the Datangpo Formation) [42,43]. In addition, a numerical model shows that high sedimentation rates result in low pyrite contents, which contrast with the high pyrite contents in the Datangpo Formation [45]. Finally, since sedimentation rates are facies-dependent and are expected to vary greatly, this model cannot explain the global superheavy pyrite formation in the Cryogenian non-glacial interval.

### Thermogenesis model

The thermogenesis model proposes a diagenetic origin of superheavy pyrite driven by thermochemical sulfate reduction (TSR) [21]. TSR is an abiotic process through which sulfate is reduced by organic matter at high temperatures [21]. Similar to MSR, TSR also generates ^32^S-enriched sulfide, and the isotopic fractionation in TSR is temperature-dependent. At temperatures >200°C, TSR is associated with ∼25‰ fractionation [51]. When TSR occurs in a closed system, the late stage Rayleigh distillation can generate ^34^S-enriched pyrite. Abundant superheavy pyrite formation through TSR requires sufficient supply of ^34^S-enriched sulfate (e.g. with the presence of evaporite) and organic matter in sediments. Thus, the thermogenesis model may explain superheavy pyrite formation in organic-rich sediments with evaporite layers above or below. Indeed, the thermogenesis model was applied to explain high pyrite content and isotopic variations in the Nanhua basin [22]. It is proposed that hydrothermal vents would release massive TSR-derived sulfide that was immediately oxidized to sulfate [22]. Vertical mixing of the ^34^S-enriched sulfate with seawater sulfate may contribute to the development of the spatial isotopic gradient of the superheavy pyrites. However, as TSR is typically a local or regional process, the applicability of this model to explain the global occurrence of superheavy pyrite in the 10-million-year Cryogenian non-glacial interval requires detailed assessment of evidence for hydrothermal activities in all Cryogenian basins.

### Marine anoxia model

Both the sulfate minimum zone (SMZ) and ocean stratification or dual-reservoir (OD) models argue for superheavy pyrite precipitation in an anoxic basin [23,47,48]. It was proposed that sulfate may have been the major oxidant in Proterozoic oceans, and thus the seawater sulfate concentration profile in the Proterozoic ocean is similar to the O_2_ concentration profile in the modern ocean. A SMZ may have developed in mid-depth seawaters, analogous to the O_2_ minimum zone in modern oceans [48]. The SMZ was characterized by extensive MSR, and thus had high δ^34^S_sw_ values [48]. Pyrite precipitated from SMZ would have high δ^34^S values [48]. Similar to the SMZ model, the OD model emphasizes the development of ocean stratification with the deep water enriched in ^34^S-sulfate [13], and pyrite precipitation from anoxic deep water would have high δ^34^S_py_ values [23]. This model can explain superheavy pyrite in sulfidic basins with low seawater sulfate levels. However, low seawater sulfate levels are inconsistent with the high pyrite contents that are typically associated with superheavy pyrite in the Cryogenian non-glacial interval. Thus, the SMZ and OD models do not provide a satisfactory explanation for the global occurrence of superheavy pyrite in the Cryogenian non-glacial interval.

### Carbon-sulfur cycle model

The carbon-sulfur (C-S) cycle model has been proposed to explain the global occurrence of superheavy pyrite in the SPICE event [18]. The SPICE event was characterized by the coupled C and S isotope positive excursions. Superheavy pyrite occurs in the climax of the SPICE event, coincident with the maximum carbonate carbon isotopes (δ^13^C_carb_) of +6‰ [18]. It is postulated that an increase of organic matter input might have stimulated MSR and enhanced pyrite burial. At a low seawater sulfate concentration, δ^34^S_sw_ was sensitive to pyrite burial, and organic carbon and pyrite burial resulted in the concurrent increases in both δ^34^S_sw_ and carbon isotope values of seawater-dissolved inorganic carbon [18]. This model is supported by the coupling of δ^13^C_carb_, δ^13^C_org_ and δ^34^S_py_. Furthermore, it is suggested that oceanic anoxia or euxinia was developed in mid-depth seawaters below the wind-mixed surface layer [18]. However, the decreasing chemostratigraphic trend of δ^34^S_py_ is associated with nearly invariant δ^13^C_org_ in the Datangpo Formation, suggesting the decoupling of C-S cycles [52].

Overall, all existing models can explain superheavy pyrite formation in the local or regional scales, but they have limited ability to explain the global occurrence of superheavy pyrite in the Cryogenian non-glacial interval. More importantly, all these models focus exclusively on the interplay between sulfate and sulfide, but ignore the importance of organosulfur compounds, which represent an important component of the modern marine sulfur cycle [27]. Below, we develop a new model that integrates organosulfur compound in sulfidic seawater to explain the global occurrence of superheavy pyrite in the Cryogenian non-glacial interval.

## A NEW MODEL FOR CRYOGENIAN SUPERHEAVY PYRITE FORMATION

Organosulfur compounds are cycled in both non-sulfidic and sulfidic conditions [28,53,54]. In a sulfidic water column, rapid organic matter sulfurization (or H_2_S methylation) can generate massive VOSC. Most VOSC is released into air where it is oxidized to SO_4_^2−^ (in the form of sulfate aerosols) largely by hydroxyl radicals in the upper atmosphere. These sulfate aerosols may be cycled back to the coastal water by precipitation or riverine influx. However, a significant amount of ^32^S-enriched VOSC or sulfate aerosols that derived from VOSC oxidation would be shuttled to the open ocean, elevating δ^34^S of sulfidic seawater in restricted anoxic marine basins. So in essence, this would drive the difference in δ^34^S_sw_ between the open ocean and restricted marginal marine basins. Here we propose that VOSC emission was at least partly responsible for the formation and distribution of Cryogenian superheavy pyrite.

Petrological and geochemical evidence indicates that most (>90%) Datangpo superheavy pyrite was precipitated within sediment porewater with H_2_S diffusion from sulfidic water column, suggesting δ^34^S_py_ values recorded δ^34^S_H2S_ near the SWI. Thus, superheavy pyrite precipitation requires high δ^34^S_H2S_ value near the SWI. Below, we develop a numerical model to simulate the development of the vertical sulfur isotope gradient in the sulfidic water column.

### Simulation of sulfur cycle in sulfidic conditions

**Figure 5.**
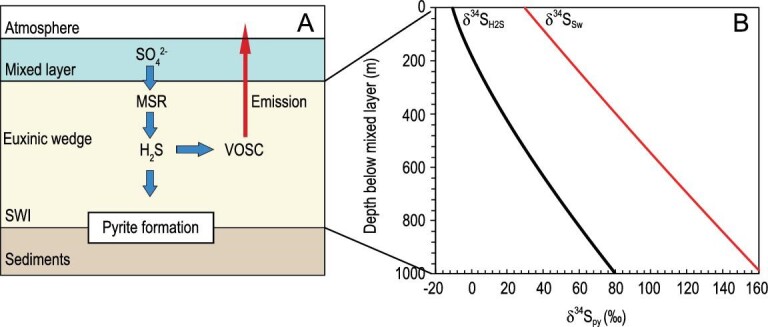
VOSC-integrated model structure and modeling results. (A) Basic model structure highlighting the contribution of VOSC to the formation of superheavy pyrite in a euxinic wedge. SWI: sediment-water interface. (B) End-member modeling results showing that VOSC emission in a stagnant ocean with a sulfidic deep-water column can drive an increase in both δ^34^S_sw_ and δ^34^S_py_ with depth. Default parameters: initial δ^34^S_sw_ value of +30‰; MSR rate constant (*k*_MSR_) at 0.0005 yr^−1^; VOSC generation rate constant (*k*_VOSC_) at 0.001 yr^−1^; seawater vertical velocity (*w*) at 0 m yr^−1^; diffusion coefficient of sulfate ($\kappa_{\rm d}$) at 0.028 m^2^ yr^−1^.

Here we use the one-dimensional diffusion-advection-reaction (1D-DAR) model to simulate the δ^34^S_H2S_ profile in sulfidic seawater on continental margins. We assume that the sulfidic layer underlies the surface mixed layer that has a homogeneous sulfate concentration and an invariant δ^34^S_sw_ value (Fig. 5A). With the presence of a large dissolved organic carbon (DOC) pool in Neoproterozoic deep ocean [52], sulfide methylation and VOSC formation were largely controlled by seawater H_2_S concentrations [55]. MSR in the sulfidic layer is sustained by sulfate diffusion from the surface mixed layer. H_2_S concentration in the sulfidic layer can be expressed as:

(7) \begin{eqnarray*} \frac{{\partial \!\left[{{{\rm{H}}_2}{\rm{S}}} \right]}}{{\partial t}} = {\rm{trans}} \!\left({{{\rm{H}}_2}{\rm{S}}} \right) + {\rm{msr}}\left( {{{\rm{H}}_2}{\rm{S}}} \right), \end{eqnarray*}

where [H_2_S] is the concentration of H_2_S, trans(H_2_S) represents the vertical transportation rate of H_2_S and msr (H_2_S) is the *in situ* change in [H_2_S] due to chemical reactions or biological activities (e.g. microbial sulfate reduction, sulfide oxidation and pyrite formation). H_2_S is generated by MSR. It is then converted to VOSC that is emitted into the atmosphere. Pyrite is mainly formed near SWI with Fe supply from sediment and H_2_S diffusion from sulfidic seawater (Fig. 5A).

The transmission term in the model can be expressed by the one-dimensional advection-diffusion equation:

(8) \begin{equation*} {\rm{trans}}\left( {\rm{X}} \right) = w \cdot \frac{{\partial \!\left[{{{\rm{H}}_2}{\rm{S}}} \right]}}{{\partial z}} + \frac{\partial }{{\partial z}}{K_{{{\rm{H}}_2}{\rm{S}}}}\frac{{\partial \!\left[{{{\rm{H}}_2}{\rm{S}}} \right]}}{{\partial z}}, \end{equation*}

where $w$ and ${K_{{{\rm{H}}_2}{\rm{S}}}}$ represent the seawater vertical velocity and vertical diffusion coefficient (the same as *D*_H_2_S_ mentioned above in equation (1)), respectively, of H_2_S.

H_2_S is generated by MSR, and the chemical reaction for MSR is as follows:

(9) \begin{equation*}{\rm{C}}{{\rm{H}}_2}{\rm{O}} + \frac{1}{2}{\rm{SO}}_4^{2 - } + {{\rm{H}}^ + } \to {\rm{C}}{{\rm{O}}_2} + \frac{1}{2}{{\rm{H}}_2}{\rm{S}} + {{\rm{H}}_2}{\rm{O}}.\end{equation*}

Assuming an unlimited supply of organic matter and invariant pH, the rate of MSR (dmsr/dt) is only controlled by the concentration of sulfate and the reaction rate constant (*k*_MSR_):

(10) \begin{equation*}{\rm{dmsr\ }} = {k_{{\rm{MSR}}}}{\rm{\ }} \cdot \left[ {{\rm{SO}}_4^{2 - }} \right] \cdot {\rm{dt}}.\end{equation*}

According to the formula of VOSC formation, with unlimited supply of organic matter, the production rate of VOSC (dvosc/dt) is a function of H_2_S concentration:

(11) \begin{equation*}{\rm{dvosc\ }} = {k_{{\rm{VOSC}}}}{\rm{\ }} \cdot \left[ {{{\rm{H}}_2}{\rm{S}}} \right] \cdot {\rm{dt}},\end{equation*}

where *k*_VOSC_ represents the rate constant of H_2_S methylation.

The time-dependent variation of seawater sulfate concentration can be calculated by:

(12) \begin{eqnarray*} \frac{{\partial \!\left[{{\rm{S}}{{\rm{O}}_4}} \right]}}{{\partial t}} + w \cdot {\rm{\ }}\frac{{\partial \!\left[{{\rm{S}}{{\rm{O}}_4}} \right]}}{{\partial z}} &=& {\kappa _{\rm d}}\ \cdot \frac{{{\partial ^2} \!\left[{{\rm{S}}{{\rm{O}}_4}} \right]}}{{\partial {z^2}}} \nonumber\\ - \, {k_{{\rm{MSR}}}}\left[ {{\rm{S}}{{\rm{O}}_4}} \right], \nonumber\\ \end{eqnarray*}

where κ_d_ is the diffusion coefficient of sulfate in seawater (the same value as the diffusion coefficient of H_2_S in seawater, see equation (8)), and *k*_MSR_ is the rate constant of MSR. At a steady state, $\frac{\partial }{{\partial t}} = {\rm{\ }}0$, we arrive at:

(13) \begin{equation*} \frac{{{{\rm{d}}^2} \!\left[{{\rm{S}}{{\rm{O}}_4}} \right]}}{{{\rm{d}}{z^2}}} - \frac{w}{{{\kappa _{\rm d}}}} \cdot \frac{{{\rm{d}}\left[ {{\rm{S}}{{\rm{O}}_4}} \right]}}{{{\rm{d}}z}} - \frac{{{k_{{\rm{MSR}}}}}}{{{\kappa _{\rm d}}}}\ \left[ {{\rm{S}}{{\rm{O}}_4}} \right] = \ 0.\end{equation*}

The boundary condition is set to:

(14) \begin{equation*} \left[ {{\rm{S}}{{\rm{O}}_4}} \right] = {C_{0,{\rm{S}}{{\rm{O}}_4}}}, \quad z = {\rm{\ }}0; \end{equation*}

(15) \begin{equation*} \left[ {{\rm{S}}{{\rm{O}}_4}} \right] = 0,\quad z \to + \infty ; \end{equation*}

where ${C_{0,{\rm{S}}{{\rm{O}}_4}}}$ is the sulfate concentration in the upper mixed layer and *z* is the depth beneath the upper mixed layer. Combining equations (13)–(15), we arrive at:

(16) \begin{eqnarray*} \left[ {{\rm{S}}{{\rm{O}}_4}} \right] &=& {C_{0,{\rm{S}}{{\rm{O}}_4}}}\! \cdot {e^{Az}}, \nonumber\\ A &=& \frac{1}{2}\ \cdot \left( {\frac{w}{{{\kappa _{\rm d}}}} - \sqrt {\frac{{{w^2}}}{{\kappa _{\rm d}^2}} + \frac{{4{k_{{\rm{MSR}}}}}}{{{\kappa _{\rm d}}}}} } \right). \end{eqnarray*}

The δ value of seawater sulfate can be calculated as:

(17) \begin{eqnarray*} {\rm{\delta }}_{{\rm{S}}{{\rm{O}}_4}}^{34} &=& \left( {\left( {\frac{{^{{\rm{34}}}{\rm{S}}{{\rm{O}}_4}\ }}{{^{{\rm{32}}}{\rm{S}}{{\rm{O}}_4}\ }}} \right)\bigg/{{\left( {\frac{{^{{\rm{34}}}{\rm{S}}{{\rm{O}}_4}\ }}{{^{{\rm{32}}}{\rm{S}}{{\rm{O}}_4}\ }}} \right)}_{\rm std}} - 1} \right)\nonumber\\ \times\, 1000\,{^{\circ}/{\circ\circ}}, \end{eqnarray*}

where ‘32’ and ‘34’ denote isotope ^32^S and ^34^S, respectively, and the subscript ‘std’ represents the standard reference of sulfur isotopes.

The time-dependent equation for H_2_S concentration is:

(18) \begin{eqnarray*} \frac{{\partial \!\left[ {{{\rm{H}}_2}{\rm{S}}} \right]}}{{\partial t}} &+& w \cdot {\rm{\ }}\frac{{\partial \!\left[ {{{\rm{H}}_2}{\rm{S}}} \right]}}{{\partial z}} = {\kappa _{\rm d}}\ \cdot \frac{{{\partial ^2} \!\left[{{{\rm{H}}_2}{\rm{S}}} \right]}}{{\partial {z^2}}}\nonumber\\ & -& {k_{{\rm{VOSC}}}} \!\left[{{{\rm{H}}_2}{\rm{S}}} \right] + {k_{{\rm{MSR}}}} \!\left[{{\rm{S}}{{\rm{O}}_4}} \right].\end{eqnarray*}

Assuming a steady state, i.e. $\frac{\partial }{{\partial t}} = {\rm{\ }}0$, we arrive at:

(19) \begin{eqnarray*} \frac{{{{\rm{d}}^2} \!\left[{{{\rm{H}}_2}{\rm{S}}} \right]}}{{{\rm{d}}{z^2}}} &-& \frac{w}{{{\kappa _{\rm d}}}} \cdot \frac{{{\rm{d}}\left[ {{{\rm{H}}_2}{\rm{S}}} \right]}}{{{\rm{d}}z}} - \frac{{{k_{{\rm{VOSC}}}}}}{{{\kappa _{\rm d}}}}\ \left[ {{{\rm{H}}_2}{\rm{S}}} \right]\nonumber\\ = \ - \frac{{{k_{{\rm{MSR}}}}}}{{{\kappa _{\rm d}}}}{C_{0,{\rm{S}}{{\rm{O}}_4}}} \cdot {e^{Az}}.\end{eqnarray*}

We set the following boundary condition:

(20) \begin{equation*} {k_{{\rm{VOSC}}}} \!\left[{{{\rm{H}}_2}{\rm{S}}} \right] = {k_{{\rm{MSR}}}} \!\left[ {{\rm{S}}{{\rm{O}}_4}} \right],\quad z\ = \ 0; \end{equation*}

(21) \begin{equation*} \left[{{{\rm{H}}_2}{\rm{S}}} \right] = \ 0,\quad z \to + \infty . \end{equation*}

The solution for equation (19) is:

(22) \begin{eqnarray*} {\rm{\ }}\left[ {{{\rm{H}}_2}{\rm{S}}} \right] &=& {{\rm{C}}_{0,{{\rm{H}}_2}{\rm{S}}}}\ \cdot {e^{Bz}} + {\left[ {{{\rm{H}}_2}{\rm{S}}} \right]_2}, \nonumber\\ B & =& \frac{1}{2}\ \cdot \left( {\frac{w}{{{\kappa _{\rm d}}}} - \sqrt {\frac{{{w^2}}}{{\kappa _{\rm d}^2}} + \frac{{4{k_{{\rm{VOSC}}}}}}{{{\kappa _{\rm d}}}}} } \right),\nonumber\\ \end{eqnarray*}

where ${{\rm{C}}_{0,{{\rm{H}}_2}{\rm{S}}}}$ is a constant, and is determined by the boundary condition; ${[ {{{\rm{H}}_2}{\rm{S}}} ]_2}$ is the particular solution for equation (19), and can be discussed in the following two scenarios:

(23) \begin{eqnarray*} {\left[ {{{\rm{H}}_2}{\rm{S}}} \right]_2} &=& \frac{{{k_{{\rm{MSR}}}}}}{{{k_{{\rm{VOSC}}}} - {k_{{\rm{MSR}}}}}}{\rm{\ }} \cdot {C_{0,{\rm{S}}{{\rm{O}}_4}}} \cdot {{\rm{e}}^{Az}},\nonumber\\ {\rm{\ }}{k_{{\rm{MSR}}}} \ne \, {k_{{\rm{VOSC}}}}; \end{eqnarray*}

(24) \begin{eqnarray*} {\left[ {{{\rm{H}}_2}{\rm{S}}} \right]_2} &=& \frac{{{k_{{\rm{MSR}}}}}}{{w - 2A \cdot {\kappa _{\rm d}}}}{\rm{\ }} \cdot {C_{0,{\rm{S}}{{\rm{O}}_4}}} \cdot z \cdot {{\rm{e}}^{Az}},{\rm{\ \ }}\nonumber\\ {k_{{\rm{MSR}}}} &=& {k_{{\rm{VOSC}}}}.\end{eqnarray*}

The vertical profile of H_2_S concentration can be expressed as:

(25) \begin{eqnarray*} \left[ {{{\rm{H}}_2}{\rm{S}}} \right] &=& {C_{0,{\rm{S}}{{\rm{O}}_4}}}\ \cdot \bigg[ \frac{{{k_{{\rm{MSR}}}}}}{{{k_{{\rm{VOSC}}}} - {k_{{\rm{MSR}}}}}} \cdot {{\rm{e}}^{Az}}\nonumber\\ \qquad\quad -\, \frac{{k_{{\rm{MSR}}}^2}}{{{k_{{\rm{VOSC}}}}\left( {{k_{{\rm{VOSC}}}} - {k_{{\rm{MSR}}}}} \right)}} \cdot {e^{Bz}} \bigg],\nonumber\\ {k_{{\rm{MSR}}}} \ne {k_{{\rm{VOSC}}}}; \end{eqnarray*}

(26) \begin{eqnarray*} \left[ {{{\rm{H}}_2}{\rm{S}}} \right] = {C_{0,{\rm{S}}{{\rm{O}}_4}}}\ &\cdot& \bigg[ \frac{{{k_{{\rm{MSR}}}}}}{{w - 2A \cdot {\kappa _{\rm d}}}} \cdot z \cdot {{\rm{e}}^{Az}}\nonumber\\ \quad +\, \frac{{{k_{{\rm{MSR}}}}}}{{{k_{{\rm{VOSC}}}}}} \cdot {e^{Bz}} \bigg],\ \nonumber\\ \qquad {k_{{\rm{MSR}}}} = {k_{{\rm{VOSC}}}}.\ \end{eqnarray*}

The δ value of seawater H_2_S can be calculated as:

(27) \begin{eqnarray*} {\delta ^{34}}\! {S_{{{\rm{H}}_2}{\rm{S}}}} &=& \left( {\left( {\frac{{^{34}{S_{{{\rm{H}}_2}{\rm{S}}}}\ }}{{^{32}{S_{{{\rm{H}}_2}{\rm{S}}}}\ }}} \right)\bigg/{{\left( {\frac{{^{34}{S_{{{\rm{H}}_2}{\rm{S}}}}\ }}{{^{32}{S_{{{\rm{H}}_2}{\rm{S}}}}\ }}} \right)}_{\rm std}} - 1} \right)\nonumber\\ \times\, 1000\,{^{\circ}/{\circ\circ}}.\end{eqnarray*}

**Figure 6.**
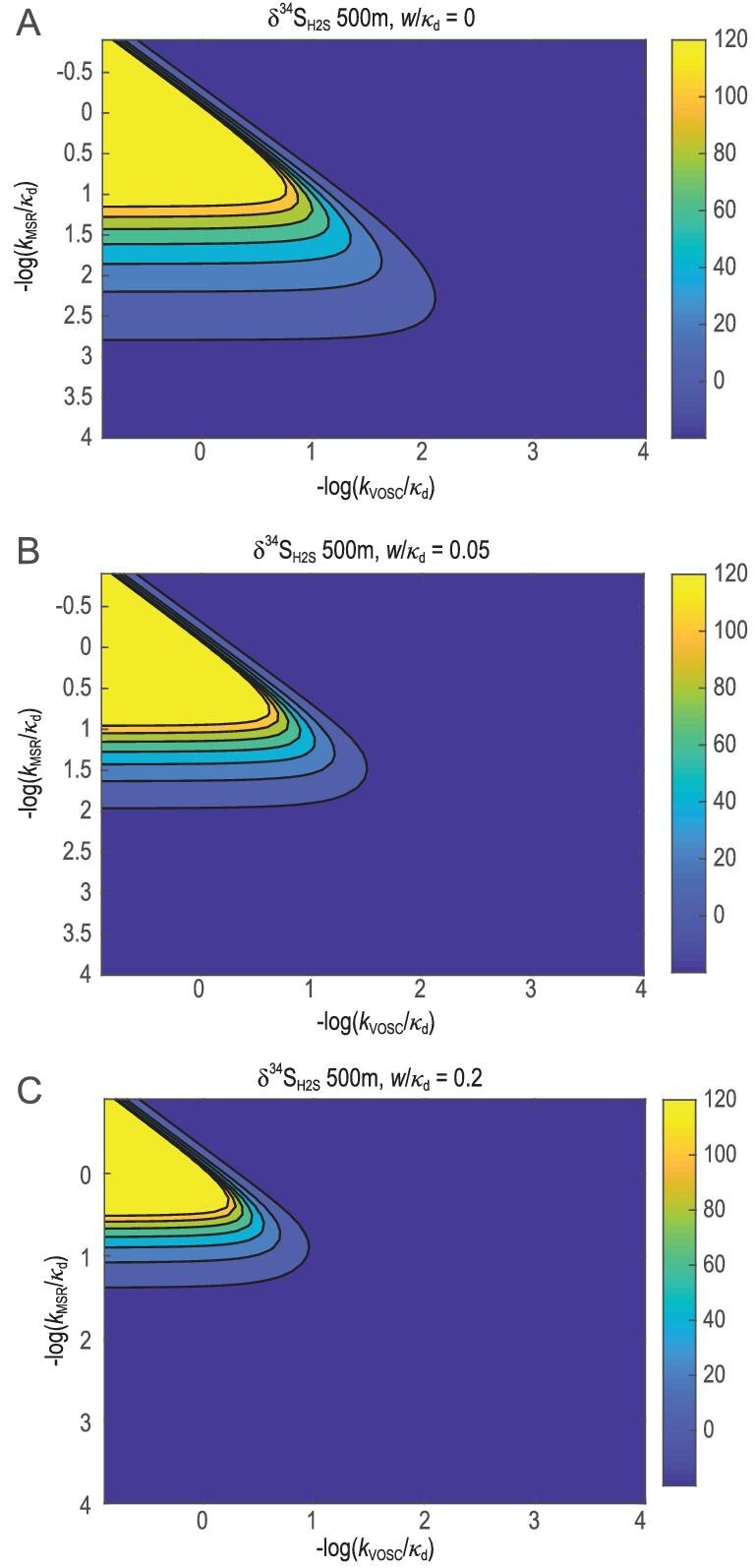
Sensitivity tests to evaluate the influence of microbial sulfate reduction rate constant (*k*_MSR_), VOSC generation rate constant (*k*_VOSC_) and vertical velocity rate (w) on δ^34^S_H2S_. The X and Y axes represent negative log of *k*_VOSC_ and *k*_MSR_, respectively, relative to $\kappa_{\rm d}$. The contour lines represent δ^34^S_H2S_. Modeling results ($\textit {w}/\kappa_{\rm d}$ = 0 m^−1^ for (A), $\textit {w}/\kappa_{\rm d}$ = 0.05 m^−1^ for (B), and $\textit {w}/\kappa_{\rm d}$ = 0.2 m^−1^ for (C)) show that superheavy pyrite formation requires a high MSR rate, high VOSC formation rate and low vertical mixing rate. Default parameters: initial δ^34^S_sw_ value of +30‰; $\kappa_{\rm d}$ = 0. 028 m^2^ yr^−1^.

Assuming the δ^34^S_sw_ value of +30‰, MSR rate constant (*k*_MSR_) at 0.0005 yr^−1^ [56] and VOSC generation rate constant (*k*_VOSC_) at 0.001 yr^−1^ [57], the model indicates that high VOSC emission can generate a large vertical δ^34^S_H2S_ gradient in sulfidic water column (Fig. 5B). It is notable that superheavy pyrite formation requires both high MSR and VOSC formation rates to maintain a strong vertical δ^34^S_H2S_ gradient (Fig. 6). In addition, a low sulfate concentration is also required for the development of such a vertical δ^34^S_H2S_ gradient, because the isotopic effect of VOSC emission could be buffered by a large sulfate reservoir. Furthermore, a low vertical mixing rate (low *w* value) or a nearly stagnant ocean is required for the sustention of the high δ^34^S_H2S_ values and strong vertical δ^34^S_H2S_ gradient.

### Global occurrence of superheavy pyrite in the Cryogenian non-glacial interval

The modeling results indicate that the formation of superheavy pyrite requires massive VOSC emission from a largely stagnant ocean to generate and maintain a strong δ^34^S_H2S_ depth gradient in sulfidic conditions. In our model, superheavy pyrite in the Datangpo Formation was mainly precipitated at or near the SWI, where both seawater sulfate and sulfide have high δ^34^S values. Our model is seemingly inconsistent with low δ^34^S_sw_ values based on carbonate-associated sulfate data (Fig. 1). However, most carbonate deposits were probably formed at shallow water depths, such that δ^34^S of carbonate associated sulfate likely reflects low δ^34^S_sw_ values of shallow waters. In contrast, higher δ^34^S_CAS_ values from authigenic Mn-carbonate in a deep-water environment [22] probably record δ^34^S_sw_ of seawaters near the SWI.

There is no direct geological or geochemical tracer for VOSC emission in paleoceans, so the VOSC model proposed here needs to be tested further. This model can be indirectly tested by evaluating the δ^34^S_H2S_ depth gradient. It predicts that (i) δ^34^S_H2S_ in the water column should be lower than δ^34^S_H2S_ at the SWI or within sediment porewater, and (ii) δ^34^S_py_ of shallow water sections should be lower than that of deep-water sections. The latter is validated by the spatial gradient in δ^34^S_py_ recorded in the Datangpo Formation: pyrite from the outer shelf sections has lower δ^34^S_py_ values than the slope sections [22].

The global occurrence of superheavy pyrite also implies widespread and massive VOSC emission during the Cryogenian non-glacial interval. VOSC emission is favored by intense MSR that generates abundant H_2_S as well as high DOC concentrations in a euxinic wedge [58]. We propose that high organic matter input might have sustained intense MSR in sulfidic seawater, which in turn fueled massive VOSC degassing [15,59–61] that, together with a reduced vertical mixing rate, created and maintained a strong δ^34^S_H2S_ depth gradient. Superheavy pyrite precipitates at the seafloor or within sediment porewater that is characterized with high δ^34^S_H2S_.

Large organic matter flux might be favored in the non-glacial interval due to the following reasons. First, deglacial intense continental weathering led to a surge of nutrient supply to the ocean, stimulating surface ocean primary productivity [59,62,63]. Second, possible occupation of empty niches in the surface ocean after the Sturtian glaciation may further enhance organic matter production [64]. Third, the transition from a cyanobacteria-dominated to an alga-dominated biological pump elevated the efficiency of organic carbon supply to the ocean interior (i.e. below the euphotic zone) [65]. Fourth, high temperature in the non-glacial interval might have weakened the thermohaline circulation in the ocean, favoring the expansion of sulfidic conditions. In fact, high surface ocean productivity is consistent with high values of carbonate carbon isotopes from the Tayshir Member in Mongolia and Hüttenberg Formation in northeastern Namibia [66] and widespread deposition of black shales in the non-glacial interval [67].

In summary, the global occurrence of superheavy pyrite may be related to extensive marine euxinia and massive VOSC degassing after the Sturtian glaciation. For the same reason, the decreasing trend of δ^34^S_py_ in the non-glacial interval (Figs 1 and 2) might be attributed to the gradual weakening of euxinic wedges, resulting in a gradual transition from sulfidic to ferruginous conditions in mid-depth seawater during the non-glacial interval [13].

### Sulfide methylation as an important process in Proterozoic marine sulfur cycle

In the modern ocean, high VOSC emission (∼1 Tmol yr^−1^) does not cause any significant perturbation in δ^34^S_sw_. VOSC in the modern ocean is mainly produced by the DMSP degradation pathway, while H_2_S methylation is quantitatively unimportant, given that sulfidic seawater covers less than 0.1% of modern seafloor [29]. H_2_S methylation is also dampened by extremely low seawater DOC concentrations, and seawater DOC is mainly composed of refractory molecules that are chemically inert [68]. Moreover, although H_2_S methylation does occur in some limited sulfidic areas, such as the Black Sea [69], the possible isotopic effect is buffered by modern high seawater sulfate concentration (∼28 mM L^−1^) [8]. In addition, VOSC is more effectively funneled back into the ocean because of high pO_2_ concentrations, and vigorous vertical oceanic mixing also homogenizes the ocean and reduces the depth gradient.

In contrast, H_2_S methylation might have played a more important role in the Proterozoic sulfur cycle, when euxinic wedges were common and seawater sulfate concentrations were low [46,58,70–72]. In a euxinic wedge, H_2_S is readily available for the formation of H_2_S-derived VOSC. In addition, a large DOC pool in the Proterozoic ocean ensures the supply of organic carbon molecules [68]. Therefore, methylation of H_2_S may be a more important process in the Proterozoic marine sulfur cycle than in the Phanerozoic.

It is notable that sulfide methylation alone does not guarantee global superheavy formation. In fact, superheavy pyrite occurred only sporadically in other geological intervals characterized by widespread sulfidic conditions, for example, during the Boring Billion (1.8–0.8 Ga, billion years ago), after the Marinoan glaciation, in the Permian-Triassic transition, and during Ocean Anoxic Events in Cretaceous [70,73]. We speculate that the absence of global superheavy pyrite precipitation after the Marinoan glaciation and in Phanerozoic may be attributed to high seawater sulfate concentrations that weakened the isotopic effects of VOSC emission and led to small δ^34^S_H2S_ depth gradients in sulfidic water column [74–76]. On the other hand, although seawater sulfate concentrations were low, relatively low primary productivity in the Boring Billion would limit MSR and accordingly reduce the δ^34^S_H2S_ depth gradient as well [77,78]. Therefore, the global occurrence of superheavy pyrite in the Cryogenian non-glacial interval may be attributed to widespread sulfidic conditions in the context of (i) the Neoproterozoic oxygenation event [13], (ii) the increase of organic matter production in the initial burst of eukaryotic primary productivity, (iii) the episodic continental nutrient supply after the Sturtian glaciation [63,65], and (iv) perhaps a more stagnant ocean.

To summarize, although additional data are needed to further test the model proposed here, our study does show that H_2_S methylation is a previously underappreciated pathway in the Proterozoic marine sulfur cycle. The interpretation of Proterozoic sulfur isotope data needs to fully consider the organosulfur cycle, which may have contributed to—if it was not solely responsible for—the sustained and global occurrence of superheavy pyrite in the Cryogenian non-glacial interval.

## METHODS

### Sulfur isotope analysis

Stable sulfur isotope analysis was performed in the Nanjing Institute of Geology and Palaeontology, Chinese Academy of Sciences. One to three grams of powder was prepared for pyrite extraction using the chromium reduction method. Pyrite-rich powder was finally converted to Ag_2_S. Dried Ag_2_S powder was mixed with 1–2 mg V_2_O_5_, and was analyzed for S isotopic compositions on a Delta V Advantage gas source mass spectrometer coupled with an Elemental Analyzer. Sulfur isotopic compositions are expressed in standard δ-notation as per mil (‰) deviations from the VCDT standard (Vienna Canon Diablo Troilite). The analytical error is <0.2‰ based on replicate analyses of samples and laboratory standards. Samples were calibrated on two international standards: IAEA-S-1: −0.3‰; IAEA-S-2: +22.7‰.

### Determination of framboidal pyrite proportion

The proportion of framboidal pyrite relative to total pyrite mass is estimated using back scattered electron images and reflected microphotographs. For each microphotograph, the proportion of framboidal pyrite can be calculated by: ${{\rm{f}}_{{\rm{fram}}}} = \frac{{{{\rm{A}}_{{\rm{fram}}}}}}{{{{\rm{A}}_{\rm{T}}}}}{\rm{\ }} \times 100{\rm{\% }}$, where A_fram_ is framboidal pyrite area in the microphotograph, and A_T_ is the total pyrite area in the microphotograph. The mean framboidal pyrite proportion in each section was calculated by: $f = \frac{{\mathop \sum \nolimits_1^n {{[{f_{\rm fram}}]}_n}}}{{\rm{N}}}$ where N is the total number of analyzed microphotographs, and *n* is the microphotograph sequence number.

## Data availability

The data that support the findings of this study are available from the corresponding author upon reasonable request.

## Supplementary Material

nwab034_Supplemental_File
