# Supplementary material for: Cracking the superheavy pyrite enigma: possible roles of volatile organosulfur compound emission
Source: Natl Sci Rev. 2021 Mar 1;8(10):nwab034. doi: 10.1093/nsr/nwab034 (PMC8566178; doi:10.1093/nsr/nwab034)
Supplement: nwab034_Supplemental_File [file nwab034_supplemental_file.docx]

**Supporting Information**

**for**

**Cracking the superheavy pyrite enigma: possible roles of volatile organosulfur compound emission**

Xianguo Lang^1,2,3^, Zhouqiao Zhao^2,4^, Haoran Ma^2^, Kangjun Huang^5^, Songzhuo Li^1^, Chuanming Zhou^3^, Shuhai Xiao^6^, Yongbo Peng^7,8^, Yonggang Liu^4^, Wenbo Tang^9^, Bing Shen^2*^

* Corresponding author (E-mail: [bingshen@pku.edu.cn](mailto:bingshen@pku.edu.cn) )

**Geological background and stratigraphic correlation**

The South China Block consists of the Yangtze Block to the northwest (present orientation) and the Cathaysia Block to the southeast (Fig. S1A). The Neoproterozoic evolution of the Yangtze Block can be divided into three stages [[1](#_ENREF_1), [2](#_ENREF_2)], after its collision with the Cathaysia Block at ca. 830 Ma to 820Ma [[1](#_ENREF_1), [3](#_ENREF_3)]. The early rifting stage is represented by the deposition of late-Tonian Liantuo/Chengjiang Formation in the inner shelf and the Banxi/Xiajiang/Danzhou Group from the outer shelf to the basin environments (Fig. S1B). The Liantuo/Chengjiang Formation is normally less than 300 m thick, whereas the Banxi/Xijiang/Danzhou Group could be several kilometers in thickness. The early rifting succession is dominated by siliciclastic deposition associated with widespread magmatic activities. Late rifting stage deposits unconformably overlies the late-Tonian deposits, and are represented by the Cryogenian (~720Ma to 635Ma) successions (Fig. S1B). Similar to the early rifting stage deposits, the Cryogenian successions are mainly composed of siliciclastic rocks, dominated by glacial marine deposits. The late rifting stage is also characterized by the quiescence of magmatism and volcanism. The Cryogenian succession thickens from <100 m in the inner shelf to several kilometers in the basinal settings (Fig. S2). The overlying Ediacaran succession, consisting of, in ascending order, the Doushantuo and Dengying/Liuchapo/Laobao formations, thins from the inner shelf (<1000 m) to the basin (<250 m) environments. The absence of volcanic and magmatic rocks in these units suggests that they represent the thermal subsidence stage of the Yangtze Block.

In the inner shelf of the Yangtze Block (such as the Yangtze Gorges area, Hubei Province), the Nantuo Formation unconformably overlies the late-Tonian Liantuo/Chengjiang Formation (Fig. S2), and can be correlated with the Marinoan glacial deposits [[4](#_ENREF_4)]. In the outer shelf of the Yangtze Block (e.g., southern Hubei and northern Hunan provinces), the Cryogenian sequence is composed of, in stratigraphic order, the Gucheng/Dongshanfeng, Datangpo, and Nantuo formations. The Gucheng/Dongshanfeng Formation unconformably overlies the late-Tonian Liantuo Formation/Banxi Group, and is composed of a thin unit (normally a few meters thick) of glacial diamictite, representing the Sturtian glacial deposits [[5](#_ENREF_5)]. The Datangpo Formation begins with a Mn-rich carbonate unit several meters in thickness, followed by a black shale unit ~10 m in thickness. The contact between the Datangpo and Nantuo formations seems gradual, and the Nantuo Formation is marked by Marinoan-age glacial diamictite. The slope sections have stratigraphic successions similar to those in the outer shelf, but the Datangpo Formation is notably thicker (>200 m). Here, the Datangpo Formation can be divided into two members: the lower member dominated by black shale and Mn-carbonate, and the upper member composed of siltstone [[6](#_ENREF_6), [7](#_ENREF_7)]. The basinal sections have the most complete Cryogenian successions in the Yangtze Block, and consist of, in ascending stratigraphic oder, the Chang’an, Fulu and Nantuo formations. In the Sanjiang area, Guangxi Province, the Fulu Formation is divided into five lithological members. Member I is composed of ironstone that is dominated by banded iron formation. Members II and IV are mainly composed of glacial diamictites, and are separated by ca.140 m thick sandstone of the member III. The uppermost of Fulu Formation (member V) is composed of ca. 20-50 m thick siltstone.

Reliable radiometric ages provide the first-order constraints on the regional stratigraphic correlation. U-Pb zircon ages of stratigraphic significance include (a) 714±8 Ma from the uppermost Liantuo Formation [[8](#_ENREF_8)], 724±12 Ma from the upper part of Liantuo Formation [[9](#_ENREF_9)], 776.6±3.8 Ma from the lower part of Liantuo Formation [[8](#_ENREF_8)], 725±10 Ma from the upper Banxi Group [[10](#_ENREF_10)], and 715.9±2.8 Ma from the uppermost Danzhou Group [[11](#_ENREF_11)]; (b) 659.96±0.46 Ma from the base of Datangpo Formation [[12](#_ENREF_12)], and 654.5±3.8 Ma from the top of Datangpo Formation [[13](#_ENREF_13)]; and (3) 635.2±0.6 Ma from the basal Ediacaran Doushantuo cap carbonate [[4](#_ENREF_4)]. Recent stratigraphic correlation suggests that member IV of the Fulu Formation can be correlated with the Gucheng/Tiesiao/Dongshanfeng Formation in slope and outer shelf settings, while the Member V can be correlated with the Datangpo Formation (Fig. S2). Such correlation is also consistent with occasional occurrence Mn-rich siltstone in member V of the Fulu Formation, which may be correlated with the Mn-carbonate in the basal Datangpo Formation.

In summary, in the Yangtze Block, the Sturtian glaciation is represented by the Chang’an and Member I to IV of the Fulu Formation in the basin environment, and the Gucheng/Tiesiao/Dongfengshan Formation in the slope and outer shelf successions, whereas the Nantuo Formation represents the deposition during the Marinoan glaciation. The Sturtian and Marinoan glaciations are separated by an interglacial interval of ~10 Myr (660–650 Ma), and the interglacial deposition is represented by the Member V of the Fulu Formation in the basin and the Datangpo Formation in the slope and outer shelf environments (Fig. S2). The two Datangpo drill cores analyzed in this study are located in Songtao County, Guizhou Province and were deposited in slope environments (Figs. S1 and S2).

**The 1D-DAR model structure**

The basic structure of the model is illustrated in figure 5A. We assume (1) all sulfate is derived from the upper mixed layer with homogeneous sulfate concentration and δ^34^S_SO4_, (2) the underlying euxinic water column is 1000 m thick, and (3) pyrite formation occurs near the water-sediment interface and/or within sediment porewater with Fe^2+^ supply from sediment porewater. In the model, the euxinic water column is equally divided into 200 layers, each 5 m thick. The upper mixed layer overlies the top layer of the euxinic water column, and there is a sediment layer below the bottom layer of the euxinic water column.

**References**

1. Wang J and Li ZX. History of Neoproterozoic rift basins in South China: implications for Rodinia break-up. *Precambrian Res*. 2003; **122**(1–4): 141–58.

2. Jiang G, Shi X, Zhang S*, et al.* Stratigraphy and paleogeography of the Ediacaran Doushantuo Formation (ca. 635–551Ma) in South China. *Gondwana Res*. 2011; **19**(4): 831–49.

3. Zhang Y, Wang Y, Zhang Y*, et al.* Neoproterozoic assembly of the Yangtze and Cathaysia blocks: Evidence from the Cangshuipu Group and associated rocks along the Central Jiangnan Orogen, South China. *Precambrian Res*. 2015; **269**: 18-30.

4. Condon D, Zhu M, Bowring S*, et al.* U-Pb ages from the Neoproterozoic Doushantuo Formation, China. *Science*. 2005; **308**(5718): 95–8.

5. Zhang QR, Chu XL and Feng LJ. Chapter 32 Neoproterozoic glacial records in the Yangtze Region, China. *Geological Society, London, Memoirs*. 2011; **36**(1): 357–66.

6. Li C, Love GD, Lyons TW*, et al.* Evidence for a redox stratified Cryogenian marine basin, Datangpo Formation, South China. *Earth Planet Sci Lett*. 2012; **331–332**: 246–56.

7. Wei W, Wang D, Li D*, et al.* The Marine Redox Change and Nitrogen Cycle in the Early Cryogenian Interglacial Time: Evidence from Nitrogen Isotopes and Mo Contents of the Basal Datangpo Formation, Northeastern Guizhou, South China. *J Earth Sci*. 2016.

8. Lan Z, Li X-H, Zhu M*, et al.* Revisiting the Liantuo Formation in Yangtze Block, South China: SIMS U–Pb zircon age constraints and regional and global significance. *Precambrian Res*. 2015; **263**: 123–41.

9. Gao W, Zhang, C.-H. . Zircon SHRIMP U-Pb ages of the Huangling granite and the tuff beds from Liantuo Formation in the Three Gorges area of Yangtze River, China and its geological significance. *Geological Bulletin of China*. 2009; **28**(1): 45-50.

10. Zhang Q-R, Li X-H, Feng L-J*, et al.* A New Age Constraint on the Onset of the Neoproterozoic Glaciations in the Yangtze Platform, South China. *J Geol*. 2008; **116**(4): 423–9.

11. Lan Z, Li X, Zhu M*, et al.* A rapid and synchronous initiation of the wide spread Cryogenian glaciations. *Precambrian Res*. 2014; **255**: 401–11.

12. Zhou C-M, Huyskens MH, Xiao S*, et al.* Refining the termination age of the Cryogenian Sturtian glaciation in South China. *Palaeoworld*. 2020; **29**(3): 462-8.

13. Zhang S, Jiang G and Han Y. The age of the Nantuo Formation and Nantuo glaciation in South China. *Terra Nova*. 2008; **20**(4): 289–94.

14. Lang X, Chen J, Cui H*, et al.* Cyclic cold climate during the Nantuo Glaciation: Evidence from the Cryogenian Nantuo Formation in the Yangtze Block, South China. *Precambrian Res*. 2018; **310**: 243–55.

**Figures and captions**


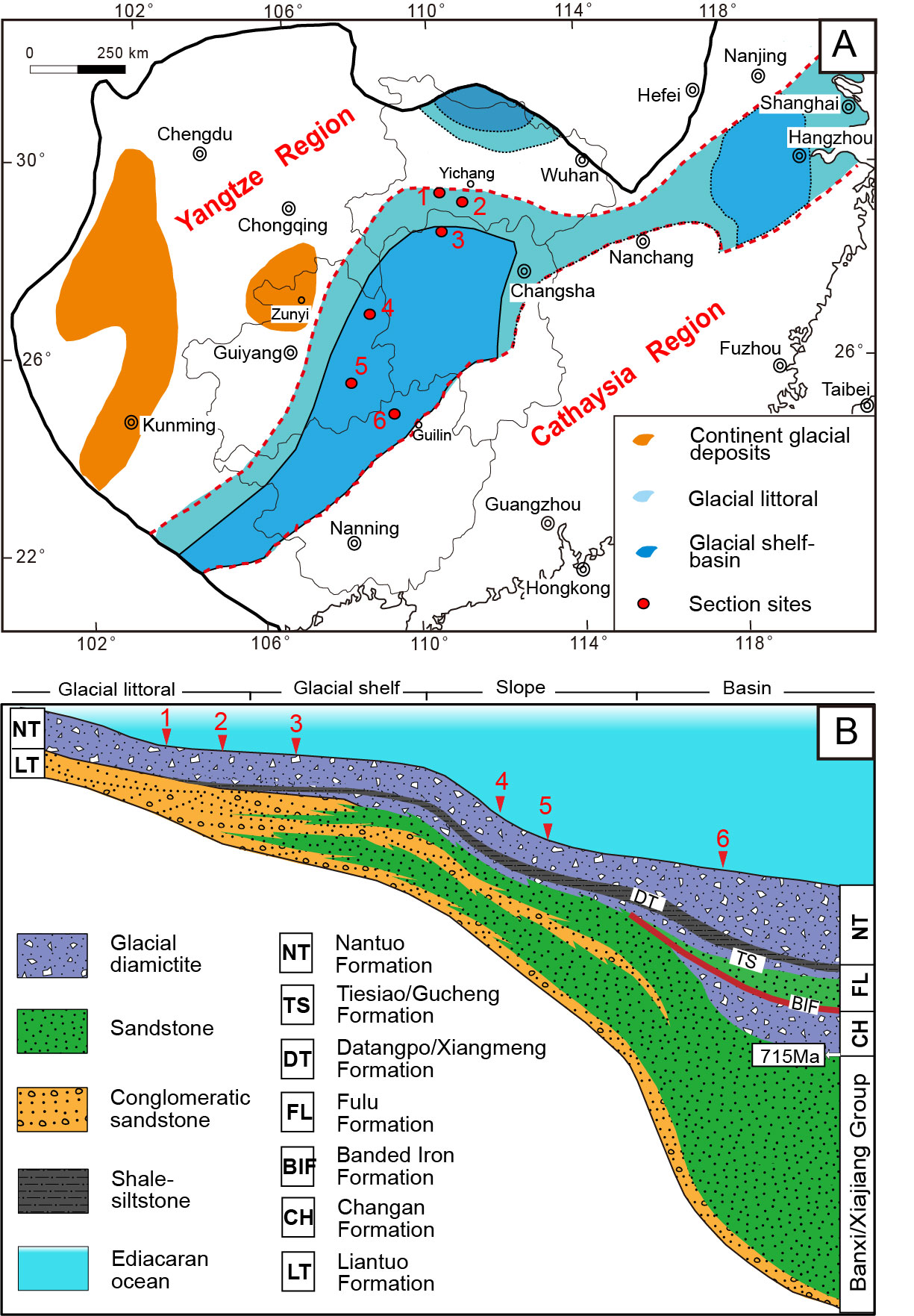


Fig.S1. Paleogeographic map and depositional model[[14](#_ENREF_14)]. **A**, Paleogeographic reconstruction of the Cryogenian Yangtze Block in South China. **B**, Cryogenian depositional model in the Yangtze Block. Red symbols denote location of stratigraphic sections: 1, Jiulongwan section; 2, Changyang section; 3, Yangjiaping section; 4, ZK-WL and ZK-DL drill cores; 5, Danzhai section; 6, Fulu section.


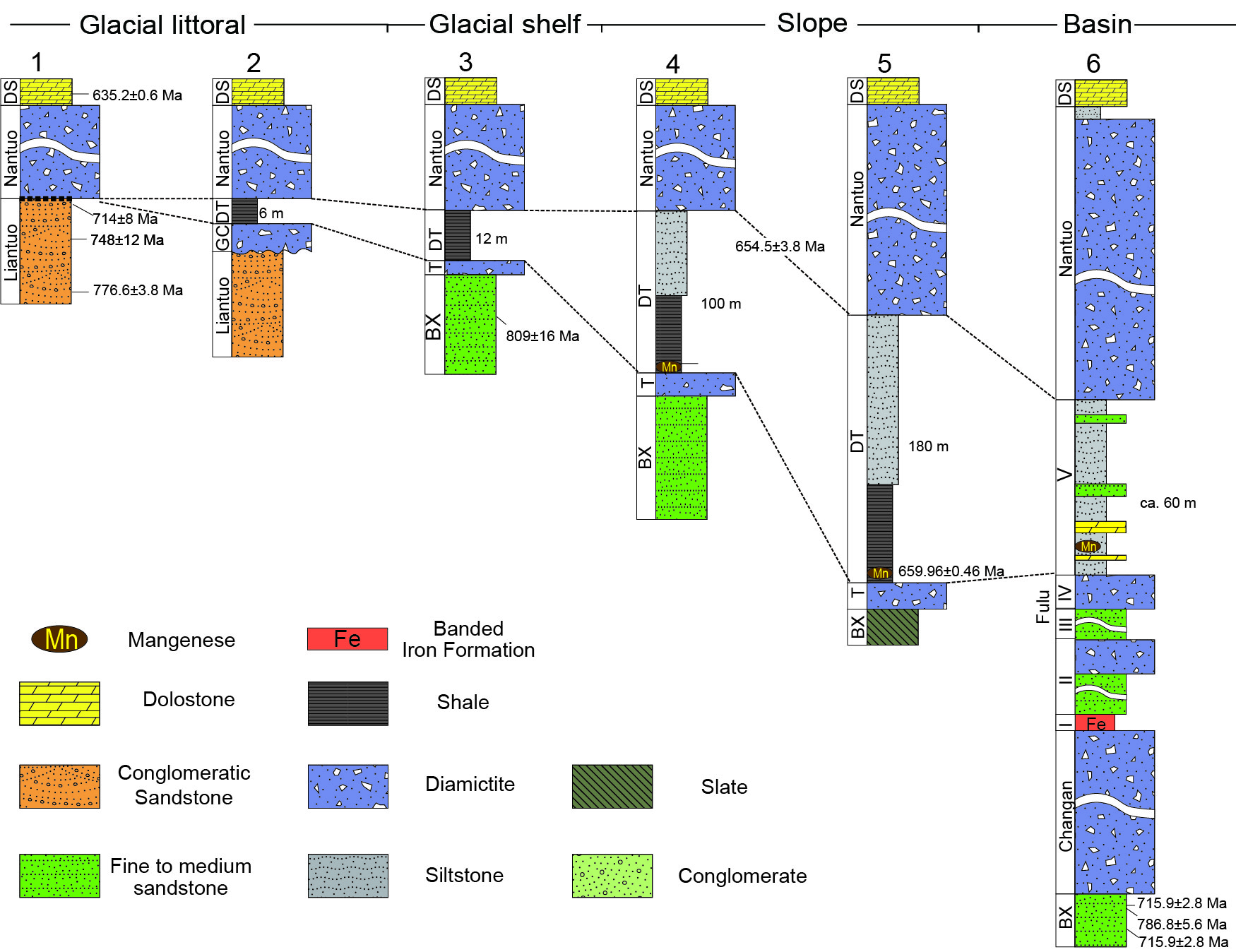


Fig. S2. Stratigraphic correlation of the representative Cryogenian sections from shelf to basin[[5](#_ENREF_5)]. Section numbers are the same as in Fig. S1. The Datangpo Formation is composed of black shale and siltstone in the slope environment. In the basinal setting, the Nantuo Formation and Fulu Formation are in conformable contact. The uppermost part of Fulu Formation (member V) is correlated with the Datangpo Formation in the slope. DS: Doushantuo Formation; DT: Datangpo Formation; GC: Gucheng Formation; T: Tiesiao Formation; BX: Banxi Group. See Supplemental Information for source of radiometric ages.


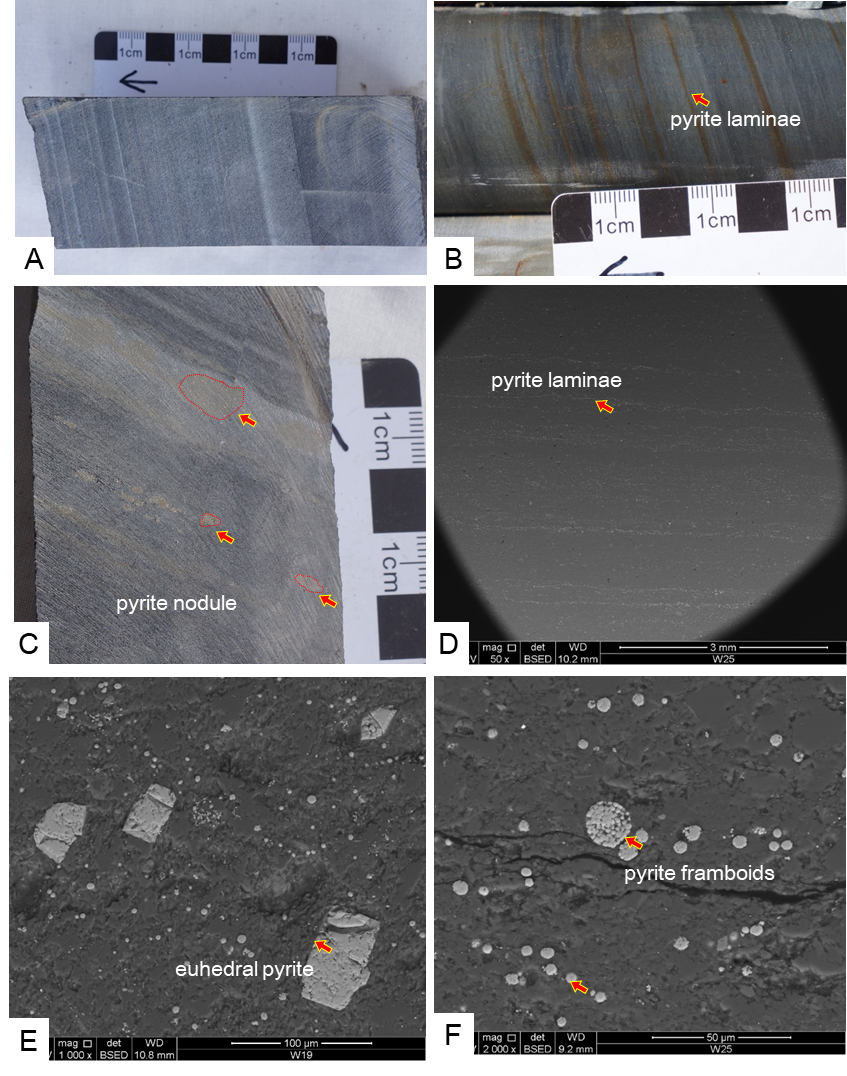


Fig. S3. Photographs of Datangpo Formation drill cores and SEM images of Datangpo pyrite. **A–C**, Photographs of Datangpo Formation drill cores showing laminated silty sandstone and shale with pyrite laminae (arrow in B) and pyrite nodules (arrows and dotted lines in C). Pyrite laminae is common in the basal Datangpo Formation. **D**, Back-scattered electron SEM micrographs showing laminae (arrow). **E and F**, Back-scattered electron SEM micrographs showing volumetrically dominant euhedral pyrite (arrow in E) and minor amount of pyrite framboids (arrows in F) in the Datangpo Formation. A, 1556 m, ZK-DL; B, 1653 m, ZK-DL; C, 1598 m, ZK-DL; D, 1608 m, ZK-DL; E, 2.5 m, ZK-WL; F, 8.2 m, ZK-WL.


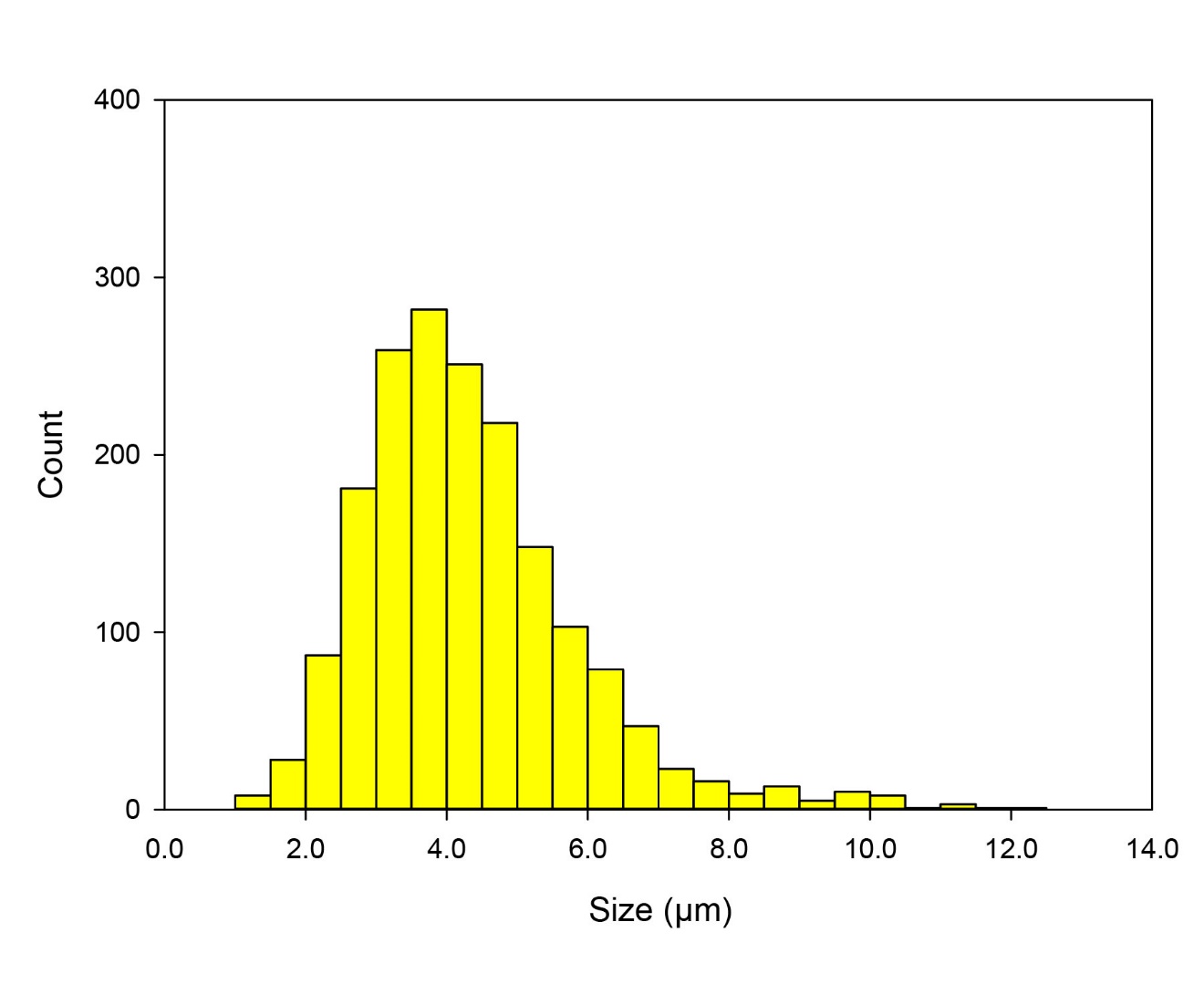


Figure S4. Size distribution of pyrite framboids from the Datangpo Formation at ZK-WL.

Table S1. Proportion of framboidal and euhedral pyrites.

| Sample NO. | Framboidal pyrite proportion | Euhedral pyrite proportion |
| --- | --- | --- |
| W1 | 4.0% | 96.0% |
| W2 | 4.7% | 95.3% |
| W3 | 17.5% | 82.5% |
| W4 | 14.1% | 85.9% |
| W5 | 5.8% | 94.2% |
| W6 | 10.9% | 89.1% |
| W7 | 9.9% | 90.1% |
| W8 | 9.9% | 90.1% |
| W9 | 11.2% | 88.8% |
| W10 | 10.2% | 89.8% |
| W11 | 10.7% | 89.3% |
| W12 | 10.9% | 89.1% |
| W13 | 12.1% | 87.9% |
| W14 | 10.4% | 89.6% |
| W15 | 8.6% | 91.4% |
| W16 | 10.3% | 89.7% |
| W17 | 9.8% | 90.2% |
| W18 | 7.7% | 92.3% |
| W19 | 6.4% | 93.6% |
| W20 | 11.6% | 88.4% |
| W21 | 9.9% | 90.1% |
| W23 | 11.2% | 88.8% |
| W25 | 16.8% | 83.2% |

Table S2. δ^34^S and pyrite content of the Datangpo Formation.

| Sample | Depth (m) | δ^34^S_py_(‰) | Pyrite content (%) |
| --- | --- | --- | --- |
| ZK-WL-1 | 0 | 58.6 | 1.9 |
| ZK-WL-2 | 0.5 | 50.1 | 2.9 |
| ZK-WL -3 | 1 | 47.9 | 0.5 |
| ZK-WL -4(R) | 1.5 | 38.1 | 5.7 |
| ZK-WL-5 | 2 | 30.4 | 4.1 |
| ZK-WL-6(R) | 2.5 | 38.1 | 4.2 |
| ZK-WL -7 | 3 | 42.3 | 3.9 |
| ZK-WL-8(R) | 3.5 | 41.8 | 3.8 |
| ZK-WL-9(R) | 4 | 38.6 | 3.8 |
| ZK-WL-10 | 4.5 | 40.8 | 4.1 |
| ZK-WL-11 | 5 | 41.3 | 2.6 |
| ZK-WL-12 | 5.5 | 42.7 | 3.6 |
| ZK-WL-13 | 6 | 35.4 | 2.7 |
| ZK-WL-14 | 6.2 | 38.3 | 3.3 |
| ZK-WL-15 | 8.2 | 55.1 | 3.6 |
| ZK-WL-16 | 9.2 | 61.9 | 3.8 |
| ZK-WL-17 | 10.2 | 60.5 | 5.8 |
| ZK-WL-18 | 11.2 | 58.4 | 5.1 |
| ZK-WL-19 | 12.2 | 56.2 | 6.4 |
| ZK-WL-20 | 13.2 | 55.2 | 3.4 |
| ZK-WL-21 | 14.2 | 53.0 | 4.2 |
| ZK-WL-22 | 15.2 | 52.2 | 5.0 |
| ZK-WL-23 | 16.2 | 40.0 | 4.0 |
| ZK-DL-6 | 1650 | 49.1 | 5.2 |
| ZK-DL-7 | 1653 | 57.8 | 6.2 |
| ZK-DL-8 | 1656 | 59.3 | 5.6 |
| ZK-DL-12 | 1662 | 56.9 | 5.8 |
| ZK-DL-14 | 1668 | 58.5 | 5.7 |
| ZK-DL-18 | 1647 | 35.8 | 3.9 |
| ZK-DL-19 | 1650 | 41.5 | 2.9 |
| ZK-DL-20 | 1641 | 41.0 | 3.8 |
| ZK-DL-22 | 1635 | 38.8 | 1.9 |
| ZK-DL-23 | 1638 | 40.1 | 1.8 |
| ZK-DL-24 | 1626 | 52.4 | 1.9 |
| ZK-DL-26 | 1621 | 53.4 | 2.9 |
| ZK-DL-28 | 1623 | 61.9 | 2.3 |
| ZK-DL-30(R) | 1614 | 19.8 | 0.1 |
| ZK-DL-31 | 1605 | 85.9 | 1.2 |
| ZK-DL-38 | 1578 | 47.8 | 0.7 |
| ZK-DL-42 | 1560 | 40.3 | 1.2 |
| ZK-DL-46 | 1550 | 28.2 | 0.3 |
